# The distribution and host range of the banana Fusarium wilt fungus, *Fusarium oxysporum *f. sp. *cubense*, in Asia

**DOI:** 10.1371/journal.pone.0181630

**Published:** 2017-07-18

**Authors:** Diane Mostert, Agustin B. Molina, Jeff Daniells, Gerda Fourie, Catur Hermanto, Chih-Ping Chao, Emily Fabregar, Vida G. Sinohin, Nik Masdek, Raman Thangavelu, Chunyu Li, Ganyun Yi, Lizel Mostert, Altus Viljoen

**Affiliations:** 1 Department of Plant Pathology, Stellenbosch University, Stellenbosch, South Africa; 2 Bioversity International–Asia Pacific, IRRI campus, Los Banos, Philippines; 3 Department of Agriculture and Fisheries, South Johnstone, Queensland, Australia; 4 Department of Microbiology and Plant Pathology, Forestry and Agricultural Biotechnology Institute (FABI), University of Pretoria, Pretoria, South Africa; 5 Indonesian Agency for Agriculture Research and Development, Jakarta, Indonesia; 6 Taiwan Banana Research Institute, Pingtung, Taiwan; 7 Lapanday Foods Corporation, Barrio Pampanga, Lanang, Davao City, Philippines; 8 Malaysian Agricultural Research and Development Institute, Selangor, Malaysia; 9 ICAR-National Research Center for Banana, Tiruchirappalli, Tamil Nadu, India; 10 Guangdong Academy of Agricultural Sciences, Institution of Fruit Tree Research, Guangzhou, Guangdong Province, China; Tallinn University of Technology, ESTONIA

## Abstract

*Fusarium oxysporum formae specialis cubense* (Foc) is a soil-borne fungus that causes Fusarium wilt, which is considered to be the most destructive disease of bananas. The fungus is believed to have evolved with its host in the Indo-Malayan region, and from there it was spread to other banana-growing areas with infected planting material. The diversity and distribution of Foc in Asia was investigated. A total of 594 *F*. *oxysporum* isolates collected in ten Asian countries were identified by vegetative compatibility groups (VCGs) analysis. To simplify the identification process, the isolates were first divided into DNA lineages using PCR-RFLP analysis. Six lineages and 14 VCGs, representing three Foc races, were identified in this study. The VCG complex 0124/5 was most common in the Indian subcontinent, Vietnam and Cambodia; whereas the VCG complex 01213/16 dominated in the rest of Asia. Sixty-nine *F*. *oxysporum* isolates in this study did not match any of the known VCG tester strains. In this study, Foc VCG diversity in Bangladesh, Cambodia and Sri Lanka was determined for the first time and VCGs 01221 and 01222 were first reported from Cambodia and Vietnam. New associations of Foc VCGs and banana cultivars were recorded in all the countries where the fungus was collected. Information obtained in this study could help Asian countries to develop and implement regulatory measures to prevent the incursion of Foc into areas where it does not yet occur. It could also facilitate the deployment of disease resistant banana varieties in infested areas.

## Introduction

The edible banana (*Musa* spp.) originated in Asia following interspecific hybridization between two species, *M*. *acuminata* Colla and *M*. *balbisiana* Colla. After domestication, seedless varieties were disseminated worldwide, and these are now grown in most tropical and some subtropical countries of the world as a fresh fruit and staple food [1, 2]. More than 150 banana varieties are grown in Asia for domestic consumption and export [3], making up 45% of all bananas grown globally [4]. India is the largest producer of bananas, with an annual production of 30 million tonnes, while China is the world’s largest producer of Cavendish bananas, with an annual production of approximately 11 million tons [5]. Other major banana producers in Asia include the Philippines (8.6 million tons) and Indonesia (5.4 million tons). Whilst most of Asia’s bananas are consumed locally, the region also produces bananas for export. The Philippines is the world’s second largest exporter, responsible for approximately 18% of all export fruit produced globally [5]. Cultivars grown on the continent differ greatly from one country to another, depending on adaptability and market preferences. India, China and the Philippines have extensive Cavendish industries, whereas countries such as Thailand, Malaysia and Indonesia grow many local varieties such as Silk and Pisang Awak [6, 7]. Production on the continent is affected by a number of biotic constraints, of which banana Fusarium wilt is one of the most devastating.

*Fusarium oxysporum* Schlecht f. sp. *cubense* (E.F. Smith) Snyder & H.N. Hansen (Foc), the causal agent of banana Fusarium wilt (Panama disease), is found in all countries where the crop is grown except those bordering the Mediterranean (apart from Egypt [8]), Somalia, and some islands in the South Pacific [9, 10]. The pathogen is believed to have co-evolved with its banana host in Asia, and from there was disseminated to new areas through infected planting material [11]. Banana Fusarium wilt is exceptionally destructive and gained infamy by disrupting the agricultural, social, economic and political landscape in Central America during the first half of the 20^th^ century [12]. Following first reports in Costa Rica and Panama in 1890, the disease rapidly spread to other Latin American countries where Gros Michel bananas were cultivated for export [1]. The disease vanished as a problem when Foc race 1-resistant Cavendish bananas were adopted to replace Gros Michel. Cavendish bananas, however, were also found to be susceptible to Foc race 4; first in the subtropics [13], and then in the tropics [14]. Because of its interaction with Cavendish bananas under different environmental conditions, Foc race 4 was divided into Foc ‘subtropical’ race 4 (SR4) and Foc ‘tropical’ race 4 (TR4) strains. The latter is particularly damaging and raised major concerns after it severely affected Cavendish plantations in Taiwan, Indonesia and Malaysia in the 1990s [14, 15, 16]. These concerns intensified when severe epidemics also occurred in Cavendish plantations in China and the Philippines [17, 18]. More recently Foc TR4 has spread to the Middle East and Africa [19]. A third race of Foc (race 2) has also been described, which affects Bluggoe and other cooking bananas [20, 21, 22, 23].

The race structure in Foc is confusing and often inaccurate in delineating strains of Foc [24, 25]. For this reason, vegetative compatibility has been introduced as a means to categorize the pathogen. Vegetative compatibility groups (VCG) in Foc are determined when the hyphae of complementary nitrate non-utilizing (*nit*)-mutants, generated on chlorate medium (CLM), anastomose to form stable heterokaryons on minimal medium (MM) [26, 27]. Isolates representing the same VCG will have the same alleles at all *vic* loci, while those in different VCGs have different alleles at one or more *vic* loci [27]. Whereas vegetative compatibility provides a clear measure of phenotypic relatedness, the technique does not measure the genetic distance between phenotypes [28, 29]. It is also labour intensive and requires approximately 2 months for identifying unknown strains.

A total of 24 vegetative compatibility groups (VCGs) have been identified for Foc, of which 21 are present in Australia and Asia [14, 23, 30, 31, 32, 33]. Some VCGs are cross-compatible and form VCG complexes, for instance VCGs 0120/15, 0124/5 and 01213/16. The largest number of Foc VCGs is found in countries where Foc is believed to have originated, such as Indonesia and Malaysia. However, VCG distribution in Asia also depends on the banana varieties grown and the prevailing climatic conditions in each country. For instance, VCGs 0120 often causes disease on Cavendish bananas in the subtropics, whereas VCGs 0121, 01213 and 01216 most commonly affect Cavendish and other diploids and triploids of *M*. *acuminata* in the tropics. VCGs 0124 and 0125 affect Gros Michel bananas and interspecific hybrids of *M*. *acuminata* and *M*. *balbisiana*, such as the AB, AAB and ABB bananas [7].

A number of DNA-based studies have been employed to determine the phylogenetic relationships between Foc VCGs. These studies all suggest that Foc is separated into two main clades and eight to ten lineages [25, 26, 27, 31, 34, 35]. Clade A affects mainly *M*. *acuminata* hybrids, whereas Clade B affects mainly *M*. *acuminata* x *M*. *balbisiana* hybrids. The lineages in Foc each contain one to five closely related VCGs [28, 29]. PCR markers have been developed to rapidly identify Foc race 4 strains [36] and the VCG complex 01213/16 (Foc TR4) [33, 37] in Clade A.

Banana Fusarium wilt occurs in all Asian countries [38, 39]. VCG diversity in Foc has been determined for strains collected in Malaysia, Indonesia, Thailand, India, China and the Philippines; all countries where a large diversity of bananas exists [28, 40, 41, 42, 43, 44, 45,46]. It is, however, the damage caused by Foc TR4 to Cavendish bananas grown in monoculture that is of particular concern to commercial growers and banana export companies [16,47]. Foc race 4 was reported in Taiwan in 1967 [20] and Foc TR4 in Indonesia and Malaysia in the 1990s [9, 48]. In the Philippines, Foc race 4 infections on Cavendish were reported as early as the 1970s [49]. The causal Foc strain was identified as VCG 0122 [50], which is regarded as less virulent to Cavendish bananas than VCG 01213/16. When Foc VCG 01213/16 began to cause large-scale epidemics to the expanding Cavendish industry in China [17] and the Philippines [18], a renewed interest and concerns of the threat of Foc to the Asian banana industry was generated. Mitigating measures are now being developed in China, the Philippines and Taiwan where Foc TR4 is causing significant losses [51]. Foc TR4 has, however, not yet been reported from India, Thailand, Sri Lanka, Bangladesh, Vietnam and Cambodia [45]. The objective of this study, therefore, was to assess the genetic diversity, distribution and host varieties affected by Foc in Asia.

## Materials and methods

### Fungal isolates

Samples collected from banana plants with Fusarium wilt symptoms in Asia from 2006 to 2007 were characterized in this study. No special permission was needed as samples were collected and submitted by individual country officers who are members of a legitimate Regional Network, the Asia Pacific Network, coordinated by Bioversity International. The species is not an endangered species. The samples were collected in China (12), Indonesia (6), Malaysia (67), the Philippines (79), Taiwan (102), Bangladesh (61), Cambodia (160), India (66), Vietnam (35) and Sri Lanka (27) (S1 Table). All collections were made during 2006 and 2007 by country representatives trained in Fusarium wilt field diagnostics, who also recorded the cultivar and locations where samples were taken. The collectors were requested to make the sampling effort as representative as possible of production areas and cultivar diversity, but this could not be guaranteed. Primary isolations were performed by plating out 5-mm pieces of infected vascular strands, which were first surface-disinfested, on potato dextrose agar (PDA) modified with 0.2% Novobiocin. Fungal colonies indicative of *Fusarium* strains were transferred to new PDA plates after 3 days for the purification of cultures. The strains were then single-spored and identified to species level.

A total of 615 *Fusarium* isolates were obtained from Asia, which are all maintained in 15% glycerol at -80°C in the culture collection of the Department of Plant Pathology at the University of Stellenbosch in South Africa (S1 Table). In addition, tester strains of the 24 VCGs of Foc were obtained from Dr Suzy Bentley and Mr Wayne O’Neill at the Queensland Department of Primary Industries in Australia, Prof Randy Ploetz from the University of Florida, Homestead FL in the USA, and Dr Kerry O’Donnell from the United States Department of Agriculture in Peoria IL in the USA (S2 Table).

### Identification of *F*. *oxysporum*

All isolates of *Fusarium* collected in Asia were single-spored and identified to species level. For cultural and morphological identification, each isolate was grown on PDA and carnation leaf agar (CLA), respectively. Colony colour and fungal morphology were then compared to characteristics of *Fusarium* spp. described by Nelson [52] and Leslie and Summerell [53]. Only isolates identified as *F*. *oxysporum* were selected for further characterization.

### DNA isolation

For DNA extraction, *F*. *oxysporum* isolates were grown in 90-mm Petri dishes on PDA at 25°C for 1 to 2 weeks. Fungal mycelium of each isolate was harvested by scraping it from the surface of the growth media with a sterile scalpel, and depositing it into Eppendorf tubes. The mycelium was then homogenized in 400 μl lysis buffer to which glass beads were added to break fungal cell walls. The Eppendorf tubes were thereafter shaken for 5 min in a Retch MM 301 shaker (Düsseldorf, Germany) and incubated at 65°C in a water bath (Polyscience, Niles, USA) for 10 min. Afterwards it was spun in a Spectrafuge 24 D centrifuge (Labnet International, Edison, USA) at 14 000 rpm for 4 min. DNA was extracted and purified using the protocol provided by Wizard SV Genomic DNA Purification System Kit (Promega, Madison, USA). The extracted DNA was quantified using a Nanodrop spectrophotometer (NanoDrop, Wilmington, USA) and stored at -20°C until further use.

### PCR and PCR-RFLP analysis

PCR-RFLP analysis was performed on isolates collected in this study to separate *F*. *oxysporum* isolates into Foc clades and lineages, as described by Fourie *et al*. 2009 [29]. Briefly, the 1500-bp intergenic spacer (IGS) region was first amplified using the primer set PNFo and PN22 [54] with an Eppendorf Mastercycler Gradient PCR machine (Eppendorf Scientific, Hamburg, Germany). The IGS region of each isolate was then subjected to restriction digests with five different enzymes to determine their clade and lineage designation. These enzymes included *Eco88 I*, *Csp6I* and *BsrD I* (Fermentas, Nunningen, Switzerland), and *BbvI* and *BceAI* (New England BioLabs, Hitchin, England). All the enzymes were used separately in PCR-RFLP digestion reactions and consisted of 10 μl IGS PCR product, 2.5 U of the restriction enzyme and 2 μl (2x) of the supplied restriction buffer. After incubation at temperatures described by enzyme specification for 3 h, the restricted fragments were separated using agarose (3%, w/v) gel electrophoresis [55]. In addition, the primer set R117 (GTC AAC CAG GAG CAG ACT G) and U9 (GTA ACC TCT GAC TCA CCG) was used to target the mitochondrial repeated (MtR) region that distinguishes between Lineage VI and VIII isolates within Clade B [29]. PCR cycling conditions consisted of 35 cycles at 94°C for 45 sec, 50°C (IGS) or 59°C (MtR) for 45 sec, and 72°C for 90 sec. Each PCR was preceded by an initial denaturation step at 94°C for 2 min and concluded by a final extension step at 72°C for 5 min. Isolates of *F*. *oxysporum* that fit the known lineages of Foc were tentatively considered members of the banana Fusarium wilt pathogen, and were selected for VCG testing. Those that did not belong to any of the known lineages were excluded from VCG analysis.

### Identification of Foc TR4 (VCG 01213/16)

To rapidly identify isolates of Foc TR4 (VCG 01213/16) in the Asian population, DNA of all isolates were amplified on an Eppendorf Mastercycler Gradient PCR machine (Eppendorf Scientific) using two sets of primers. These included FocTR4-F and FocTR4-R developed by Dita *et al*. (2010) [33], as well as VCG 01213/16 F1 and VCG 01213/16 R2 developed by Li *et al*. (2013) [37]. The PCR assay was conducted using 20 ng of fungal DNA in a total volume of 25 μl containing 1x Buffer, 2 mM MgCl_2_, 1 U Taq DNA polymerase, 0.32 mM dNTPs, 0.8 mg.ml^-1^ BSA and 0.2 μM of each primer. PCR cycle conditions for the Foc TR4 primer set consisted of 30 cycles at 95°C for 1 min, 60°C for 1 min and 72°C for 3 min. Each PCR was preceded by an initial denaturation step at 95°C for 5 min and concluded by a final extension step at 72°C for 10 min. The cycling conditions for the VCG 01213/16 primer set consisted of 35 cycles at 94°C for 45 sec, 64°C for 45 sec and 72°C for 60 sec. Each PCR was preceded by an initial denaturation step of 94°C for 5 min and concluded with a final extension step of 72°C for 5 min. As a positive control, a known VCG 01213/16 isolate (CAV 789) was included, as well as a non-template control.

### Nit-mutant generation and VCG testing

*Nit*-mutants of wild type Foc isolates were generated on minimal media (MM) amended with 1.5–3.0% KClO_3_ and incubated at 25°C for 7–21 days, as described by Puhalla (1985) [26]. Spontaneous KClO_3_-resistant sectors that developed were transferred to MM slants. Those that grew as thin colonies with no aerial mycelium were classified as *nit*-mutants and were further characterized on media containing one of four different sources of nitrogen [56]. The VCG identity of all the Asian Foc isolates were then determined by pairing *nit*-1 and *nit*-3 mutants of the Asian isolates with Nit-M testers of known VCGs testers. A VCG identity was assigned to an unknown Foc isolate if a heterokaryon was formed between its *nit*-1 or *nit*-3 mutant and the known tester’s Nit-M mutants [57]. *Nit*-1 mutants of Foc isolates that were not assigned any known VCG identity were paired with their own Nit M mutants to test for heterokaryon incompatibility. Once intra-strain compatibility was established, they were tested for compatibility with isolates that were also incompatible with tester strains representing known VCGs. All pairings were repeated at least once. The mutants are all maintained on MM slants at 4°C at the facilities of the Department of Plant Pathology, Stellenbosch University.

## Results

### Morphological identification of *F*. *oxysporum*

Of the 615 isolates investigated in this study, 594 had cultural and morphological characteristics typical of *F*. *oxysporum* (Table 1) [52, 53]. Microconidia were produced in false heads on short monophialides and were mostly single-celled and kidney-shaped. The microconidia were produced in abundance, and thin, sickle-shaped macroconidia were produced in moderate amounts. Chlamydospores were formed singly and sometimes in pairs with a coarse protective wall after 4 weeks on CLA, and in some cases only after 6 weeks of incubation. The colour of colonies differed from cream to peach, cream to dark purple and cream to purple-peach. Twenty-one of the isolates were identified as other species of *Fusarium* (S1 Table) and excluded from further analysis.

**Table 1:** Vegetative compatibility group (VCG) and lineage distribution of *Fusarium oxysporum* f. sp. *cubense* isolates in Asia.

Clade	A				B								U^1^		
Lineage	I/II	IV	V		VI				VII			U^1^	U^1^		
VCG	0126	0122	0121	01213/16	0124/5	0128	01220	0124/22	0123	01217	01218	01221	NC^2^	SI^3^	Total
**China**				11									1		**12**
**Indonesia**				4	1				1						**6**
**Malaysia**			1	40	2	1			4	3	1		10	2	**64**
**Philippines**	2	2		24					24				15	6	**73**
**Taiwan**			3	92	1	1							2		**99**
**Bangladesh**					35	2	10	1	1	2			8		**59**
**Cambodia**					117	2		2	6	2	1	14	10		**154**
**India**					50	1	3	1					10		**65**
**Sri Lanka**					13		1			1			11	1	**27**
**Vietnam**					27	2		1	1			1	2	1	**35**
**Total**	**2**	**2**	**4**	**171**	**246**	**9**	**14**	**5**	**37**	**8**	**2**	**15**	**69**	**10**	**594**

^1^PCR-RFLP fingerprint not corresponding to Clade or Lineage identity as reported by Fourie *et al*. (2009) [29]

^2^Isolates not compatible to known VCGs

^3^Self-incompatible isolates

### Molecular identification of Foc

Of the 594 *Fusarium oxysporum* isolates collected from wilted bananas in Asia, 179 isolates were divided into Clade A (30%) and 336 isolates into Clade B (57%) respectively, based on their PCR-RFLP profiles (Table 1). Seventy-nine isolates (13%) did not fit into either of the two clades, and were excluded from further analysis in this study. Forty-one Clade A isolates were collected in Malaysia (64% of total isolates from country), four in Indonesia (67%), 95 in Taiwan (96%), 28 in the Philippines (38%) and 11 in China (92%), respectively. Fifty-one Clade B isolates were collected from Bangladesh (86%), 144 collected from Cambodia (94%), 32 collected from Vietnam (91%), 55 collected from India (85%), and 15 collected from Sri Lanka (56%). The majority of isolates in Clade A (Lineages I-V) represented Lineage V (96%), whereas Clade B (Lineages VI-VIII) isolates represented Lineages VI and VII. Of the latter, Lineage VI contained 84% of the isolates. All known VCGs showed the expected PCR-RFLP fingerprint according to Fourie *et al*. (2009) [29]. VCGs 0121 and 01213/16 were in Lineage V, VCGs 0124/5, 0128, 01212, 01220 and 01222 in Lineage VI and VCGs 0123, 01217 and 01218 in Lineage VII (Table 1). VCG 01221 was the exception. While showing to be in Clade B, it did not correspond to PCR-RFLP fingerprints of Lineage VI, VII or VIII (Table 1).

The primer sets developed for VCG 01213/16 identification by Dita *et al*. (2010) [33] and Li *et al*. (2013) [37] amplified a 463-bp and a 455-bp [33, 37] band, respectively. None of the isolates representing VCGs other than VCG 01213/16 produced amplification products with either of the primer sets.

### Nit mutant generation and VCG testing

*Nit* mutants were successfully generated for all the Asian Foc isolates. Ninety-nine percent of isolates produced both Nit-M and *nit*-1 or *nit-*3 mutants, which were used for compatibility testing. Ten isolates were self-incompatible and did not produce heterokaryons when their Nit-M and *nit*-1 mutants were paired (Table 1) [27]. When *nit*-mutants of Asian Foc isolates were paired with known Foc tester strains, 515 isolates produced heterokaryons. In total, 14 of the 24 known VCGs were identified in Asia, with those representing the 0124/5 and 01213/16 VCG complexes dominating (Table 1). Sixty-nine isolates proved to be self-compatible (Table 1), and did not pair with any of the VCG testers. They were thus excluded from further analysis.

### VCG diversity in Asia

For a comprehensive analysis of Foc diversity and host interaction throughout Asia, VCG and host data of 80 Foc isolates from China [37], 117 isolates from Thailand [45], and 47 isolates from Indonesia [58] published recently, were combined with results from the current study. Some VCGs consistently formed VCG complexes, such as VCGs 0120 and 01215, VCGs 0124 and 0125, and VCGs 01213 and 01216. Results for these VCGs were therefore combined.

**Fig 1:**
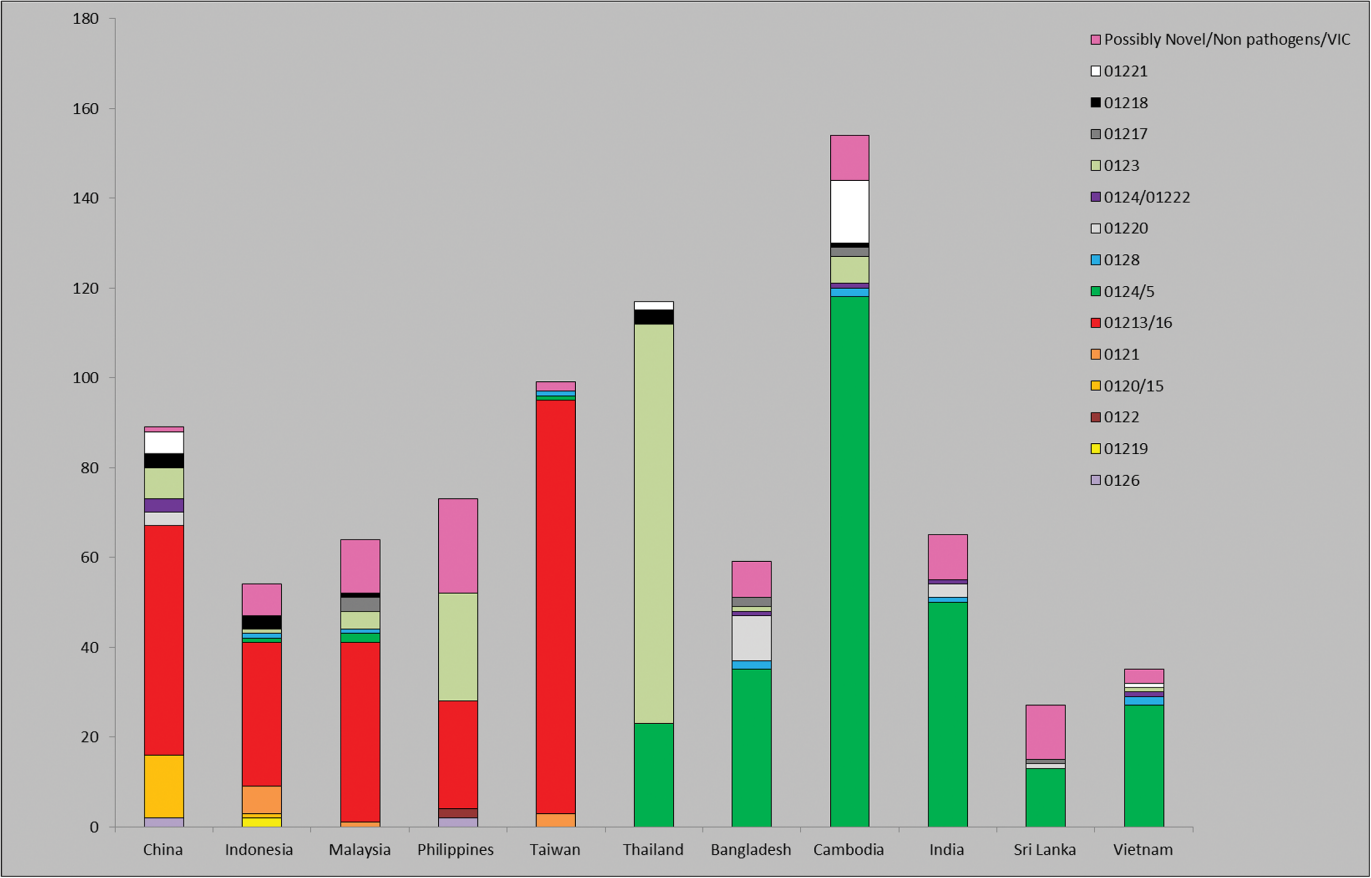
Distribution of vegetative compatibility groups of *Fusarium oxysporum* f. sp. *cubense* found in Asian countries. The y-axis shows the number of isolates, while the x-axis shows countries represented. The legend corresponds each of the VCGs to a specific colour: VCG 0120/15 (light orange), 0121 (dark orange), 0122 (burgundy), 0123 (light green), 0124/5 (dark green), 0126 (light purple), 0128 (blue), 01213/16 (red), 01217 (dark grey), 01218 (black), 01219 (yellow), 01220 (light grey), 0124/22 (dark purple) and self-incompatible and isolates incompatible to known VCGs (pink).

Countries in Asia with the greatest number of known VCGs were mainland China (8), Indonesia (7) and Cambodia (7) (Fig 1). The country with the least amount of VCGs recorded was Sri Lanka, with three VCGs. The VCG complex 01213/16 dominated in Southeast Asia including China, Indonesia, Malaysia, the Philippines and Taiwan, whereas VCG complex 0124/5 was dominant in the Indian subcontinent including India, Bangladesh, Sri-Lanka, Cambodia and Vietnam. Some VCGs were limited to specific countries, for instance VCG 01219 in Indonesia and VCG 0122 in the Philippines (Fig 1).

#### VCGs in the Philippines

**Fig 2:**
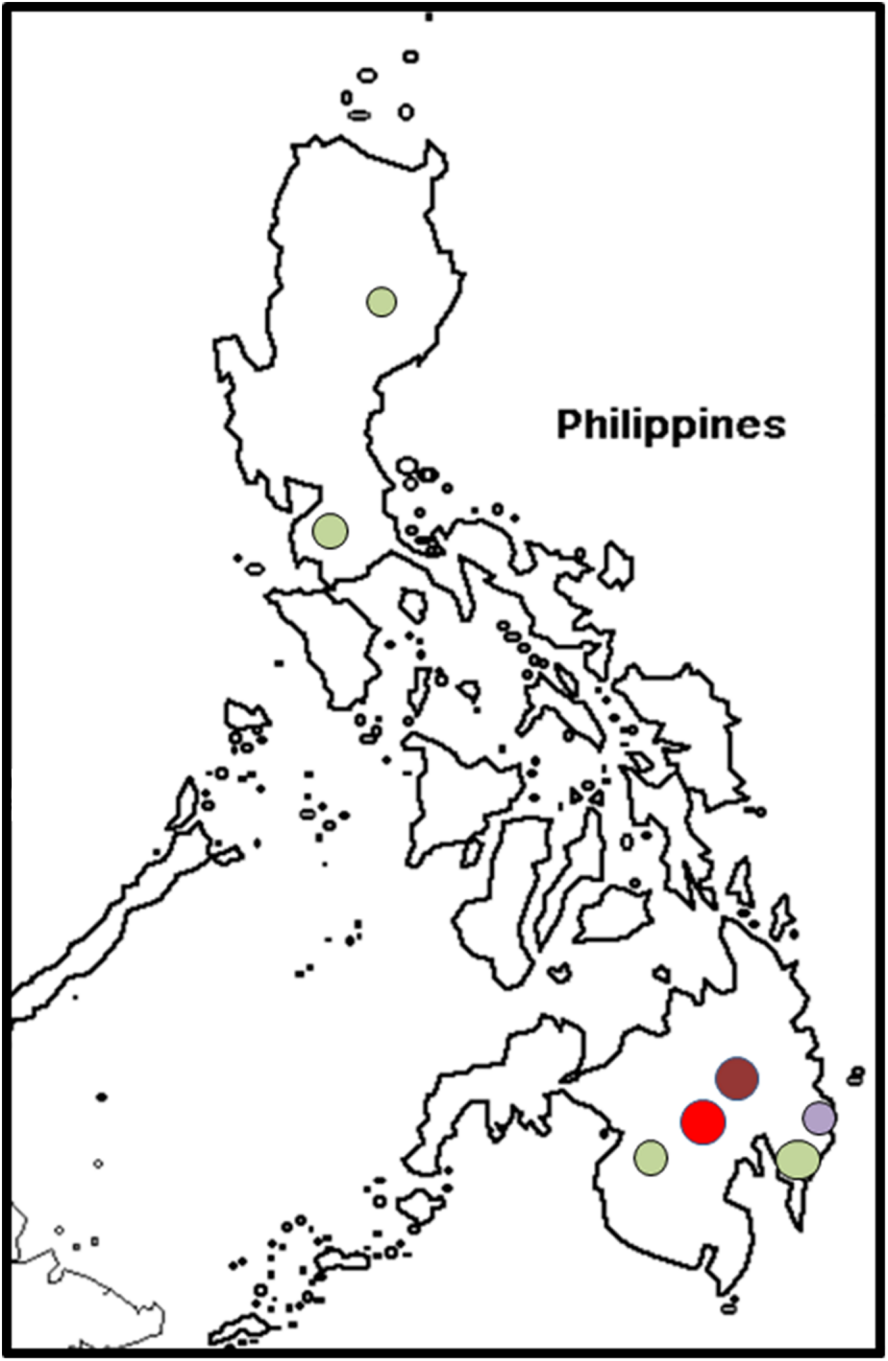
The distribution of *Fusarium oxysporum* f. sp. *cubense* (Foc) vegetative compatibility groups (VCGs) in the Philippines. VCG 0122 is shown in maroon, VCG 0123 is shown in green, VCG 0126 is shown in light purple and VCG 01213/16 is shown in red.

Fusarium wilt in Cavendish bananas around Davao on the southern Philippine island of Mindanao was caused by Foc VCG 01213/16 (Fig 2). VCG 0123 was found both on the Mindanao and Luzon islands, and was isolated mainly from the Silk cultivar Latundan (AAB) (S1 Table). VCG 0122 was associated with the Cavendish cultivar Grande Naine (AAA) in the Davao del Norte area, whereas VCG 0126 was only found in the Comval province (Mindanao) on the Saba cultivar Cardaba (ABB) (Fig 2). Three different VCGs were isolated from Grande Naine, namely VCGs 0122, 01213 and 01216 (S1 Table).

#### VCGs in Taiwan

**Fig 3:**
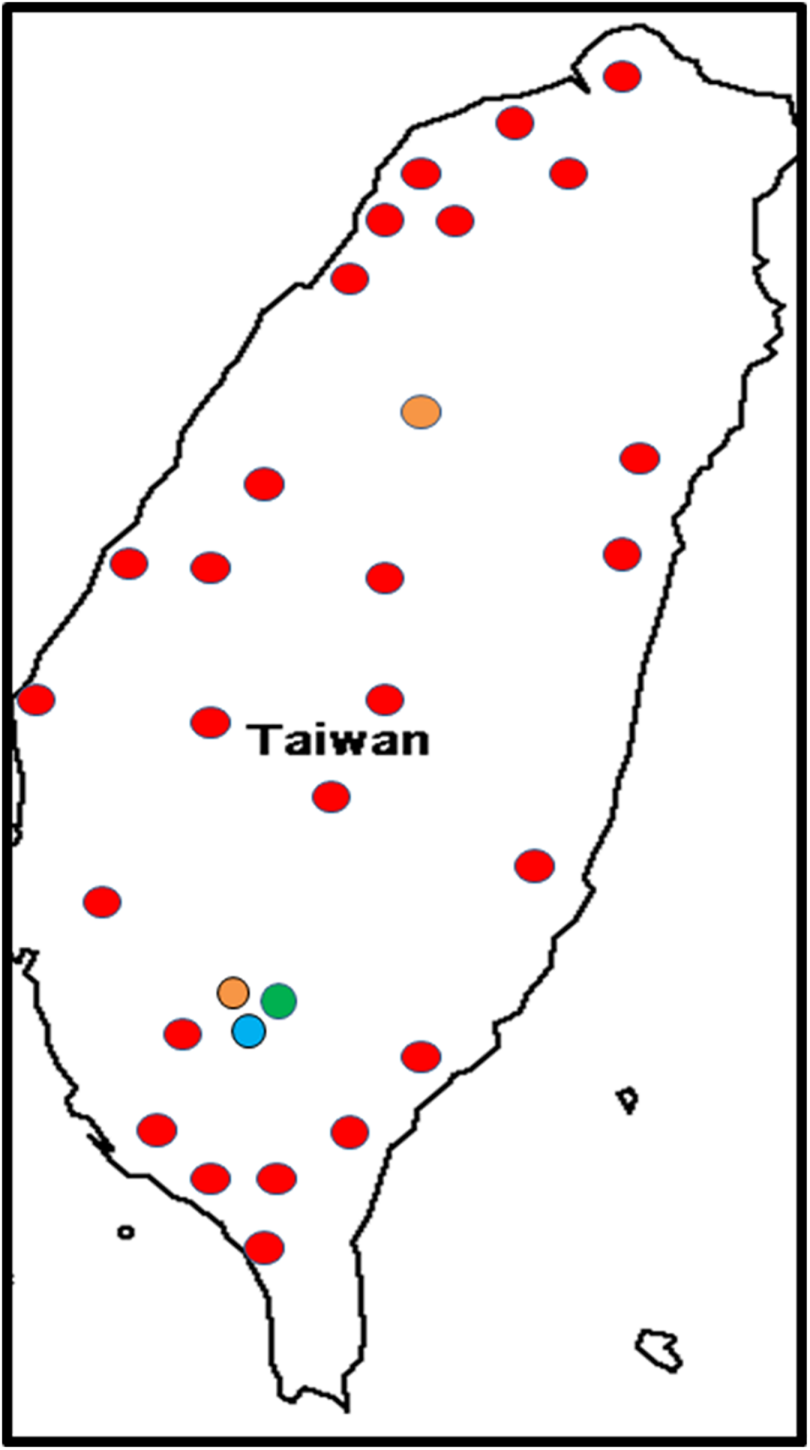
The distribution of *Fusarium oxysporum* f. sp. *cubense* (Foc) vegetative compatibility groups (VCGs) in Taiwan. VCG 0121 is shown in light orange, VCG 0124/5 is shown in green, VCG 0128 is shown in blue and VCG 01213/16 is shown in red.

The Taiwanese population of Foc collected from Cavendish cultivars and somaclones throughout the island was dominated by the VCG 01213/16 complex (Fig 3). Other VCGs isolated included VCGs 0121, 0124 and 0128. VCG 0121 was found in Kuosin and Tsatsen on the Pisang Awak cultivar Namwa (ABB) and on Latundan (Fig 3). Only one isolate of VCGs 0124 and 0128 each were isolated from the Namwa (S1 Table) in Mingchian. The banana cultivar affected by most VCGs in Taiwan was Namwa, which was affected by VCGs 0121, 0124, 0128, 01213/16 and an unidentified *F*. *oxysporum* isolate (S1 Table). A second unidentified *F*. *oxysporum* isolate was found in the Cavendish cultivar Pei-Chiao (Giant Cavendish).

#### VCGs in Malaysia

**Fig 4:**
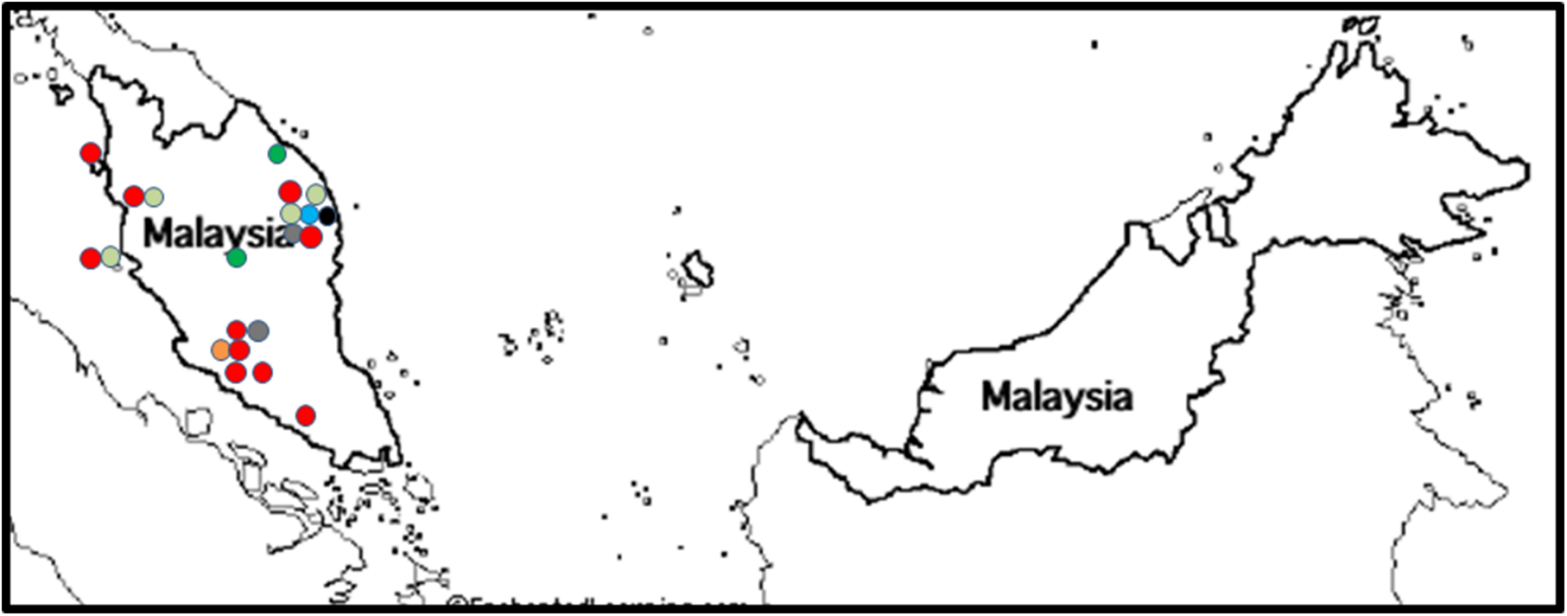
The distribution of *Fusarium oxysporum* f. sp. *cubense* (Foc) vegetative compatibility groups (VCGs) in Malaysia. VCG 0121 is shown in light orange, VCG 0123 is shown in light green, VCG 0124/5 is shown in dark green, VCG 0128 is shown in blue, VCG 01213/16 is shown in red, VCG 01217 is shown in black and VCG 01218 is shown in dark grey.

Foc was collected from bananas in Malaysia from the western Malay Peninsula, but not from Malaysian Borneo (Fig 4). The VCG 01213/16 complex was widespread (Fig 4) and found on six cultivars affected by Fusarium wilt (S1 Table). These included the Lakatan cultivar Pisang Berangan (AAA), the Sucrier cultivar Pisang Mas (AA), Pisang Raja (AAB), Pisang Awak (ABB), the Bluggoe cultivar Pisang Abu Keling (ABB) and Port Dickson (genome unknown) (Table 2). Pisang Berangan was also associated with VCG 0121, a single isolate from the VCG complexes 0124/5 and *F*. *oxysporum* isolates not compatible with known VCGs (S1 Table). Four other VCGs were found in Malaysia. VCGs 01217 and 01218 were found on the Silk banana Pisang Rastali (AAB) and the Bluggoe cultivar Pisang Abu Keling (ABB) in northern Peninsular Malaysia, VCG 0123 on Pisang Awak and Pisang Rastali in the northeast and northwest of Peninsular Malaysia, and VCG 0128 in the Kelantan area (Table 1; Fig 4), for which no cultivar information was available. Twelve unidentified isolates of *F*. *oxysporum* were obtained from banana in Malaysia. Two of these were VCG incompatible, while 10 isolates incompatible to known VCG testers were associated with Mas, Pisang Kapas, Pisang Abu Keling, Pisang Berangan, Pisang Awak, Pisang Rastali and Plantain (S1 Table).

**Table 2:** The group, subgroup and common synonyms of *Musa* cultivars and their relationship with vegetative compatibility groups (VCGs) of *Fusarium oxysporum* f. sp. *cubense*.

Group	Subgroup	Cultivar	Synonyms ^1^	VCG association
AA	Sucrier	Mas Kirana	Emas, Amas, Pisang Mas, Kluai Khai, Chuoi Trung	01213/16, N^2^
	Inarnibal	Manih		N
	Other^3^	Rejang	Rose	01213/16
AAA	Cavendish	Giant Cavendish	Pei-Chiao	01213/16, N
			Formosana^4^, Tai-Chaio #3^4^	01213/16
		Ambon Hijau	Buai	0121, 0122, 01213/16
		Grande Naine	Pisang Ambon Jepang, Chuoi Va Huong	0122,0123, 01213/16, N
		Brazilian	Baxi	0120/15, 0123, 01213/16, 01221
	Gros Michel	Ambon Kuning	Ambon, Pisang Embun, Kluai Hom Thong, Chuoi Tieu Cao #2	0124/5, 01213/16, N
	Lakatan	Berangan	Pisang Berangan Kuning, Kluai Hom Maew, Pisang Berangan Merah, Kluai Ngang Phaya	0121, 0124/5, 01213/16, N
	Ibota	Pisang Kapas	Yangambikm 5, Alaswe	N
	Red	Red	Chevvazhai, Morado, Pisang Raja Udang Merah, Pisang Udang, Kluai Nak, Chuoi Com Lua	0124/5, N
AB	Ney Poovan	Champa	Poovan	0124/5, 0128, 01217, 01220, N
AAB	Other	Dajiao		01218
	Plantain	Plantain		0124/5, 01220, N
	Pome	Hill Banana	Virupakshi, Sirumalai, Malaivazhai	N
	Pisang Raja	Raja	Kluai Khai Boran #2, Radja	01213/16, N
	Mysore	Mysore	Inangel, Pisang Keling, Kluai Lanka, Chuoi Com Chua	01213/16
	Silk	Latundan	Rasthali, Rasabale, Malbhog, Mortaman, Pisang Rastali,Pisang Raja Serai, Kluai Nam, Chuoi Goong, Loka Dadi, Raja Serai	0121, 0123, 0124/5, 01213/16, 01217,N
		Sabri	Rasthali	0123, 0124/5, 01217, 01220, 0124/22, N
		Bangla Kola		0124/5
	Laknau	Pisang Panjang		01213/16
ABB	Pisang Awak	Kluai Namwa	Awak, Pisang Awak, Kluai Namwa, Chuoi Su, Chuoi Tay, Guangfen, Siem, Karpooravalli	0120/15, 0121, 0123, 0124/5, 0126, 0128, 01213/16, 01217, 01218, 01220, 01221, 0124/22, N
	Bluggoe	Chuoi Ngop	Pisang Abu Keling, Kluai Nom Mi, Chuoi Ngop Lun	0124/5, 01213/16, 01218, 01220,N
	Ney Mannan	Ney Mannan		0124/5, 01220
	Monthan		Chuoi Ngop Cau, Kluai Nom Mi, Pisang Abu Bujal	0124/5,N
	Saba	Cardaba	Kepok, Pisang Nipah, Pisang Kepok, Kluai Hin	0120/15, 0126, 01213/16, 01219, N
BB	Other	Manohar		0128, N
Other	Other	Loka Pumbu		01213/16
		Port Dickson		01213/16

^1^Synonyms from Valmayor *et al*. (2000) [3]

^2^N, refers to isolates not compatible to known VCGs

^3^“Other” refers to unknown genotype or subgroup identity

^4^Somaclones selected from Giant Cavendish

#### VCGs in Indonesia

**Fig 5:**
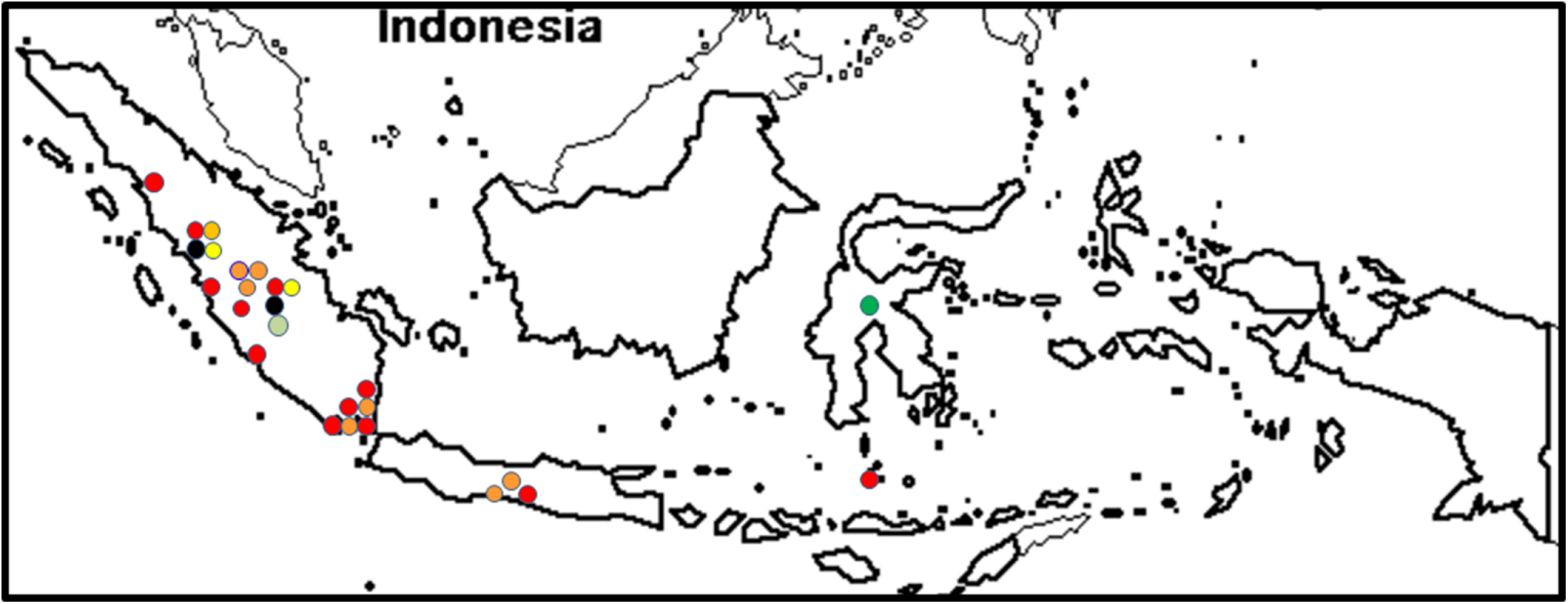
The distribution of *Fusarium oxysporum* f. sp. *cubense* (Foc) vegetative compatibility groups (VCGs) in Indonesia. VCG 0120/15 is shown in dark yellow, VCG 0121 is shown in light orange, VCG 0123 is shown in light green, VCG 0124/5 is shown in dark green, VCG 01213/16 is shown in red, VCG 01218 is shown in black and VCG 01219 is shown in light yellow.

Seven Foc isolates collected from Java, Sumatra and Sulawesi in Indonesia were identified in this study. More isolates from western Sumatra were identified by Riska and Hermanto (2012) [58]. The Foc population in Indonesia was highly diverse, and included VCGs 0120/15, 0121, 0123, 0124/5, 0128, 01213/16, 01218 and 01219 (S1 Table) [58]. The VCG complex 01213/16 was most widespread (Fig 5), and was isolated from the local banana cultivars Loka Dadi and Loka Pumbu, and from Pisang Raja (AAB), the Silk banana cultivar Pisang Raja Serai (AAB), Pisang Barangan (AAA), the Gros Michel cultivar Ambon Kuning (AAA), the Cavendish cultivars Ambon Hijau and Buai (AAA), the Rose banana cultivars Rejang (AA) and Jantan, the Saba cultivar Pisang Kepok (ABB), the Sucrier cultivar Pisang Mas Kirana (AA) and the Laknau cultivar Pisang Panjang (AAB) (Table 2) [58]. Most VCGs in Indonesia were isolated from Kepok [58] and Loka Dadi [S1 Table]. The isolates from West Sumatra included VCGs 0120, 0121, 01213/16 and 01219, with VCG 01219 found in two regions, Dharmasraya and Solok (Fig 5) [58]. The local cultivar Loka Dadi was associated with VCGs 0123, 0124 and 01213/16 (S1 Table).

#### VCGs in China

**Fig 6:**
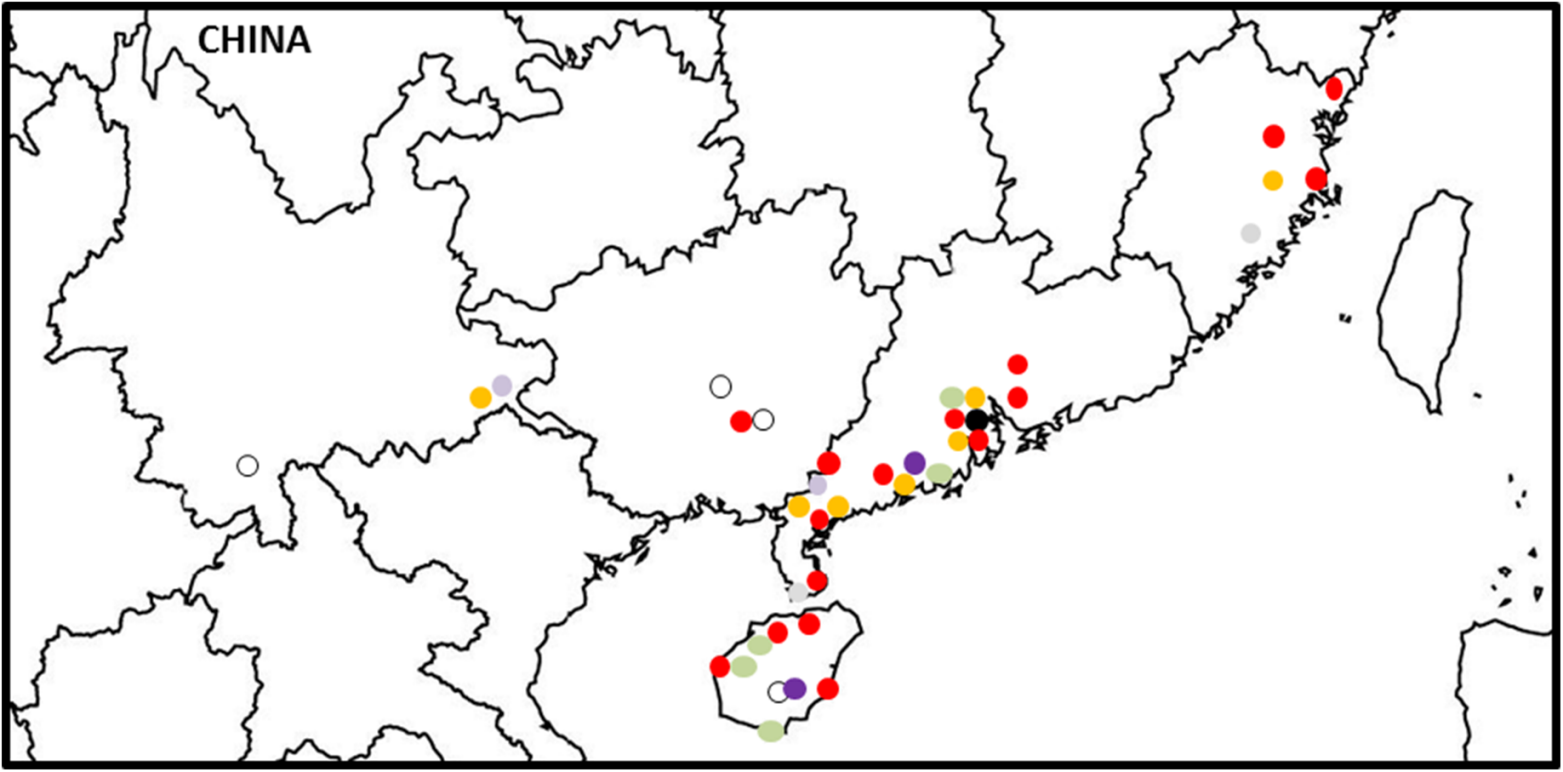
The distribution of *Fusarium oxysporum* f. sp. *cubense* (Foc) vegetative compatibility groups (VCGs) in China. VCG 0120/15 is shown in dark yellow, VCG 0123 is shown in light green, VCG 0126 is shown in light purple, VCG 01213/16 is shown in red, VCG 01218 is shown in black, VCG 01220 is shown in light grey, VCG 01221 is shown in white and VCG 0124/22 is shown in dark purple.

The greatest diversity of Foc in Asia was found in China. Collections were conducted in five different banana-producing provinces by Li *et al*. (2013) [37]. Additionally, 5 isolates from the Cavendish cultivar Brazilian (AAA) and the Pisang Awak cultivar Guangfen (ABB) and six isolates with no cultivar information were identified as VCG 01213/16 in the current study (S1 Table). Other VCGs identified in China included VCGs 0120/15, 0123, 0126, 01218, 01220, 01221 and 0124/01222. Guangdong Province hosted the largest and most diverse population of Foc, including the VCG complexes 0120/15, 01213/16 and 0124/22, as well as VCGs 0123, 0126 and 01220 (Fig 6). VCG 01213/16 complex was widespread in all production areas, except for Yunnan Province. VCG 01218 was isolated in the Guangdong province only. Most VCGs in the Chinese population were associated with Guangfen, including VCGs 0120/15, 0123, 0124/22, 0126, 01213/16, 01220 and 01221 (S1 Table) [37]. Da Jiao, an AAB variety, was only affected by VCG 01218 (Table 2) [37].

#### VCGs in Bangladesh

**Fig 7:**
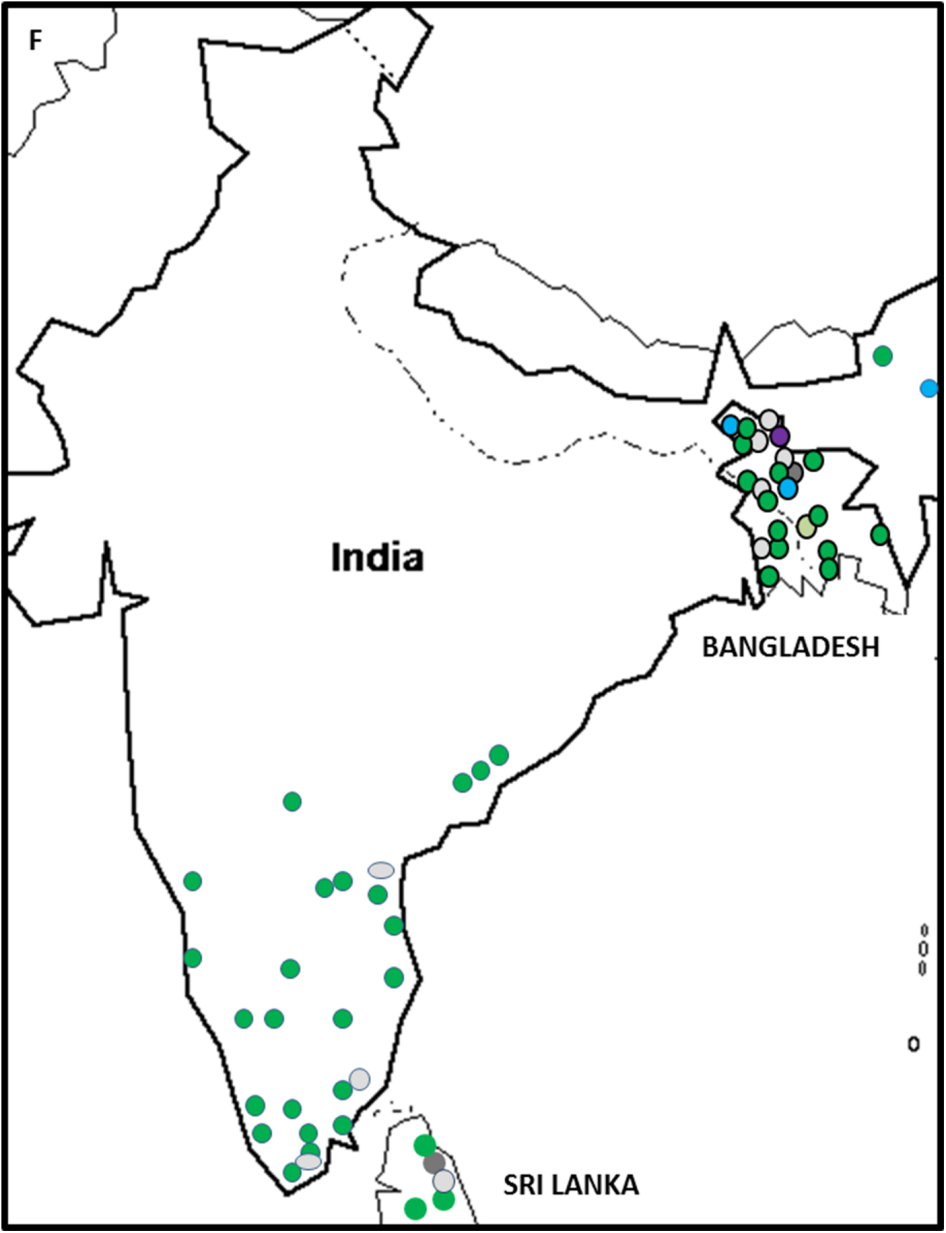
The distribution of *Fusarium oxysporum* f. sp. *cubense* (Foc) vegetative compatibility groups (VCGs) in Bangladesh, India and Sri Lanka. VCG 0123 is shown in light green, 0124/5 is shown in dark green, VCG 0128 is shown in blue, VCG 01217 is shown in dark grey, VCG 01220 is shown in light grey and VCG 0124/22 is shown in purple.

Foc was found widespread throughout Bangladesh (Fig 7). The population was dominated by the VCG 0124/5 complex, which was obtained from all affected banana cultivars in the country; including the Silk type banana Bangla Kola, the AAB bananas Sabri and Plantain, and the AB Ney Poovan banana Champa (Table 2). VCG 01220 was isolated from Sabri and Plantain in western Bangladesh along the border with India (Fig 7). The single isolate of VCG 0123 was found in central Bangladesh and VCG 0124/01222 in northern Bangladesh. VCG 0128 was isolated in the northwest and VCG 01217 in the north-central region (Fig 7). The cultivar most affected by Fusarium wilt in Bangladesh was Sabri, which was associated with all the VCGs found in the country with the exception of VCG 0128. It was also affected by an isolate not compatible to known VCGs (S1 Table).

#### VCGs in India

**Fig 8:**
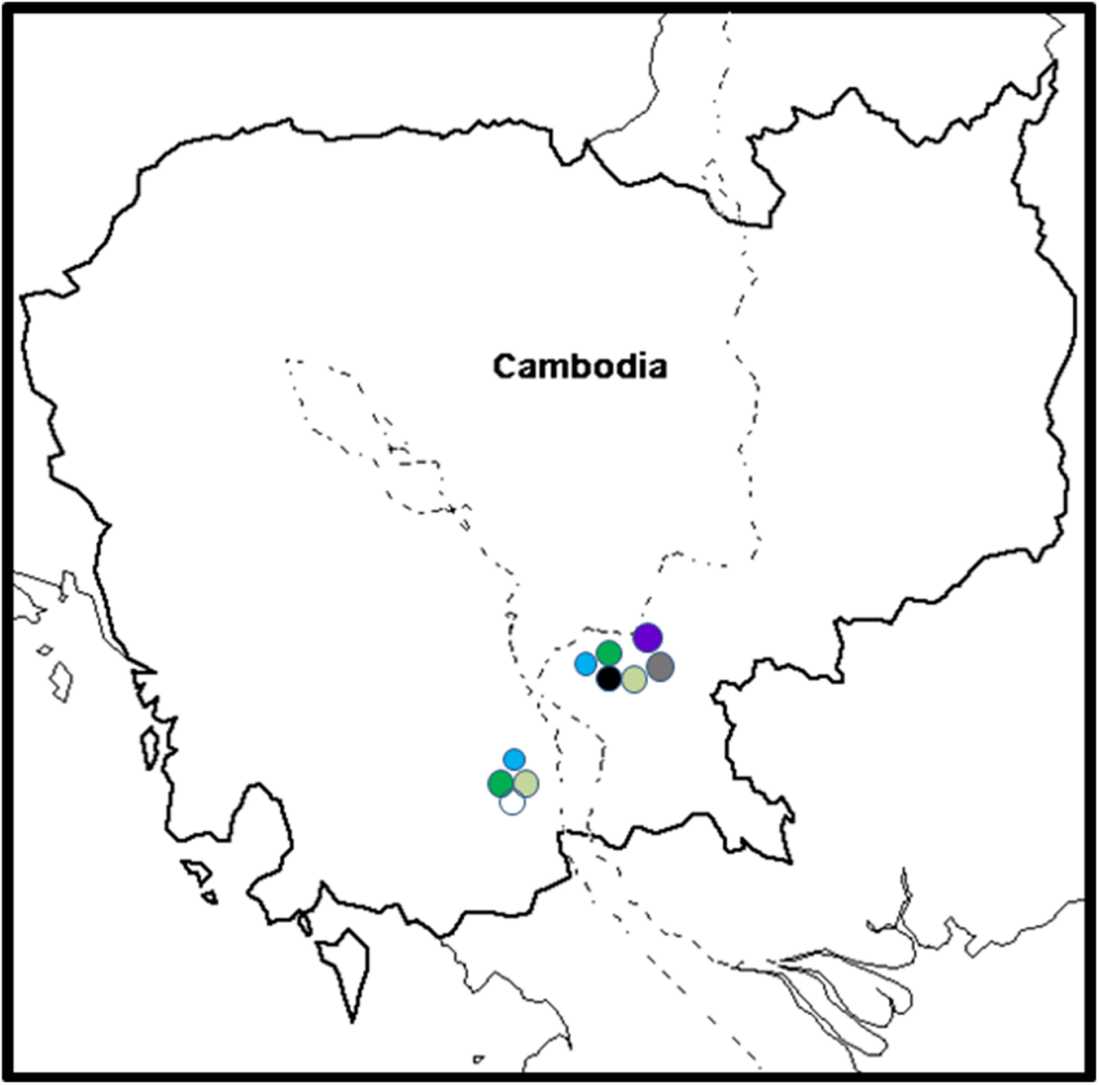
The distribution of *Fusarium oxysporum* f. sp. *cubense* (Foc) vegetative compatibility groups (VCGs) in Cambodia. VCG 0123 is shown in light green, VCG 0124/5 is shown in dark green, VCG 0128 is shown in blue, VCG 01217 is shown in dark grey, VCG 01218 is shown in black, VCG 01221 is shown in white and VCG 0124/22 is shown in purple.

Banana Fusarium wilt was detected in several important banana varieties grown across India (Fig 8). The dominant VCG found in India was the VCG 0124/5 complex (Fig 1), and isolates from this VCG were collected from ABB bananas including Monthan and the Pisang Awak cultivar Karpooravalli, AB bananas such as Ney Poovan, and the Silk cultivars Rasthali, Malbhog and Mortaman (AAB) (Table 2). VCG 0128 isolated from BB cultivar Manohar, was found in a single location north on the border next to Myanmar (Fig 7). A number of banana cultivars were also affected by isolates of Foc not fitting into existing VCGs. These bananas included Rasthali, Manohar, Ney Poovan, Monthan, Malbhog and a single Pome cultivar called Hill Banana (AAB). The cultivars from which most VCGs were collected in India were Karpooravalli (S1 Table); which were associated with VCGs 0124/5, 01220 and the VCG complex 0124/22; and Ney Poovan associated with VCGs 0124/5 and VCG 01220 (S1 Table). No isolates were collected from Cavendish bananas, which are planted widely in India.

#### VCGs in Sri Lanka

The relatively small Sri Lankan population of Foc composed primarily of isolates representing VCG 0124/5 (Fig 1). These were isolated from a range of cultivars in the central and Sabaragamuwa provinces, including Gros Michel (AAA), Red (AAA), Pisang Awak, Silk and Ney Mannan cultivars. Only one isolate of VCG 01217 from Silk and one VCG 01220 isolate from a Ney Mannan (ABB) cultivar were collected in Sri Lanka (S1 Table). No location information was available for these isolates. Ten isolates collected from Gros Michel, Silk and Red cultivars in the central, western and Sabaragamuwa provinces did not belong to any known VCG of Foc (S1 Table, Fig 7).

#### VCGs in Cambodia

This study provides the first population study based on VCG identification of Foc from Cambodia (Table 1). Isolation was only done from Pisang Awak cultivar Namwa (S1 Table). Two general areas were affected: Kampong Cham and Kandal. Kampong Cham (south-eastern part of Cambodia) were most severely affected, and all VCGs identified in the country (VCGs 0123, 0124/5, 0124/22, 0128, 01217, 01218, and 01221) were found in this area. Only VCGs 0123, 0124/5, 0128 and 01221 were found in Kandal (Fig 8).

#### VCGs in Thailand

**Fig 9:**
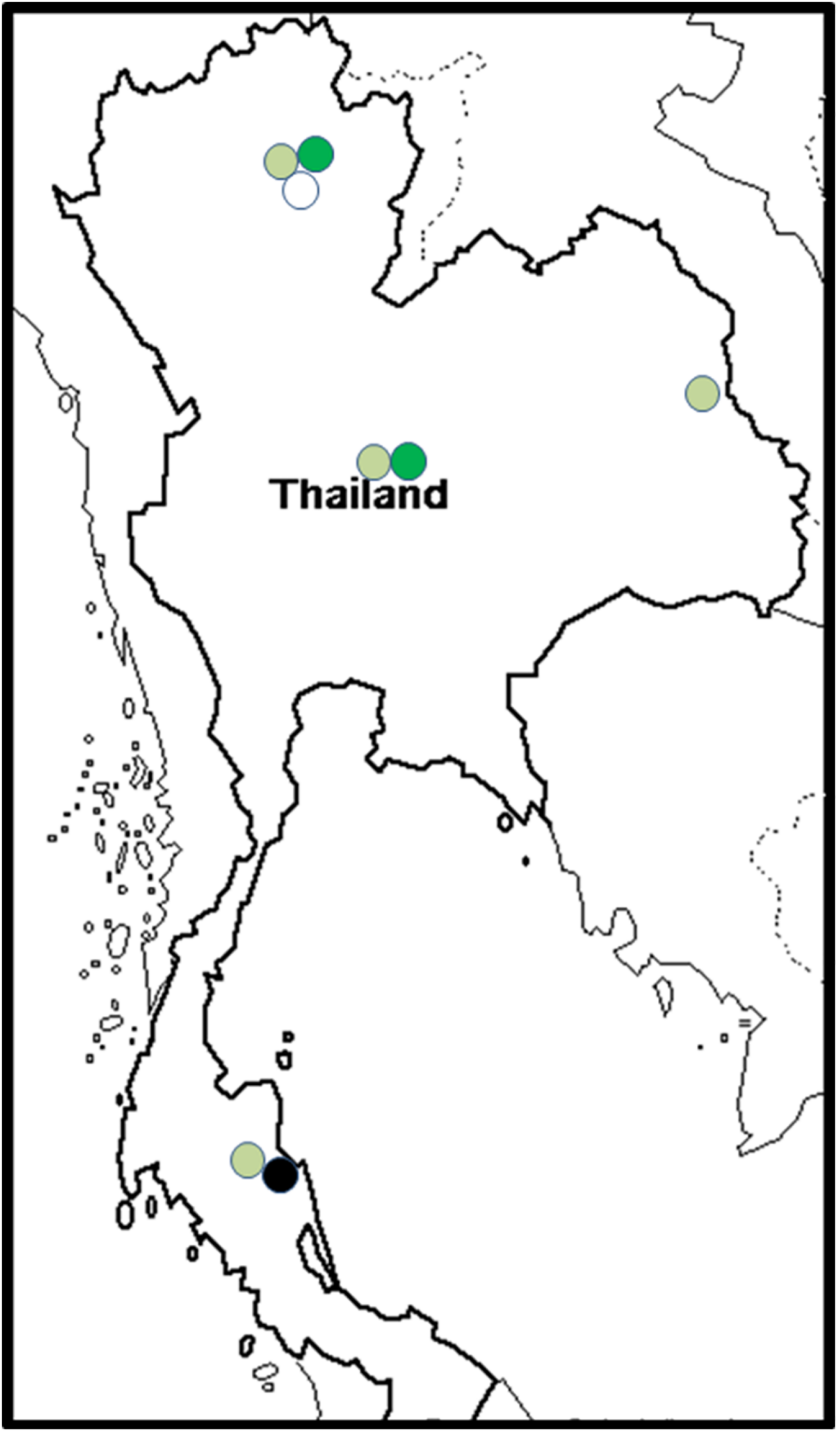
The distribution of *Fusarium oxysporum* f. sp. *cubense* (Foc) vegetative compatibility groups (VCGs) in Thailand. VCG 0123 is shown in light green, 0124/5 is shown in dark green, VCG 01218 is shown in black and VCG 01221 is shown in white.

Thailand was the only country in Asia where the Foc population was not dominated by either the VCG 0124/5 or VCG 01213/16 complexes [45]. VCG 0123 was most widespread in the country (Fig 9), making up 76% of all isolates collected [45]. All Foc isolates were collected from the ABB Pisang Awak cultivar Kluai Namwa [45]. The VCGs complex 0124/5 was found in the northern and central part of Thailand, whereas VCG 01221 was restricted to the north (Fig 9). VCG 01218 was isolated in the south [45].

#### VCGs in Vietnam

**Fig 10:**
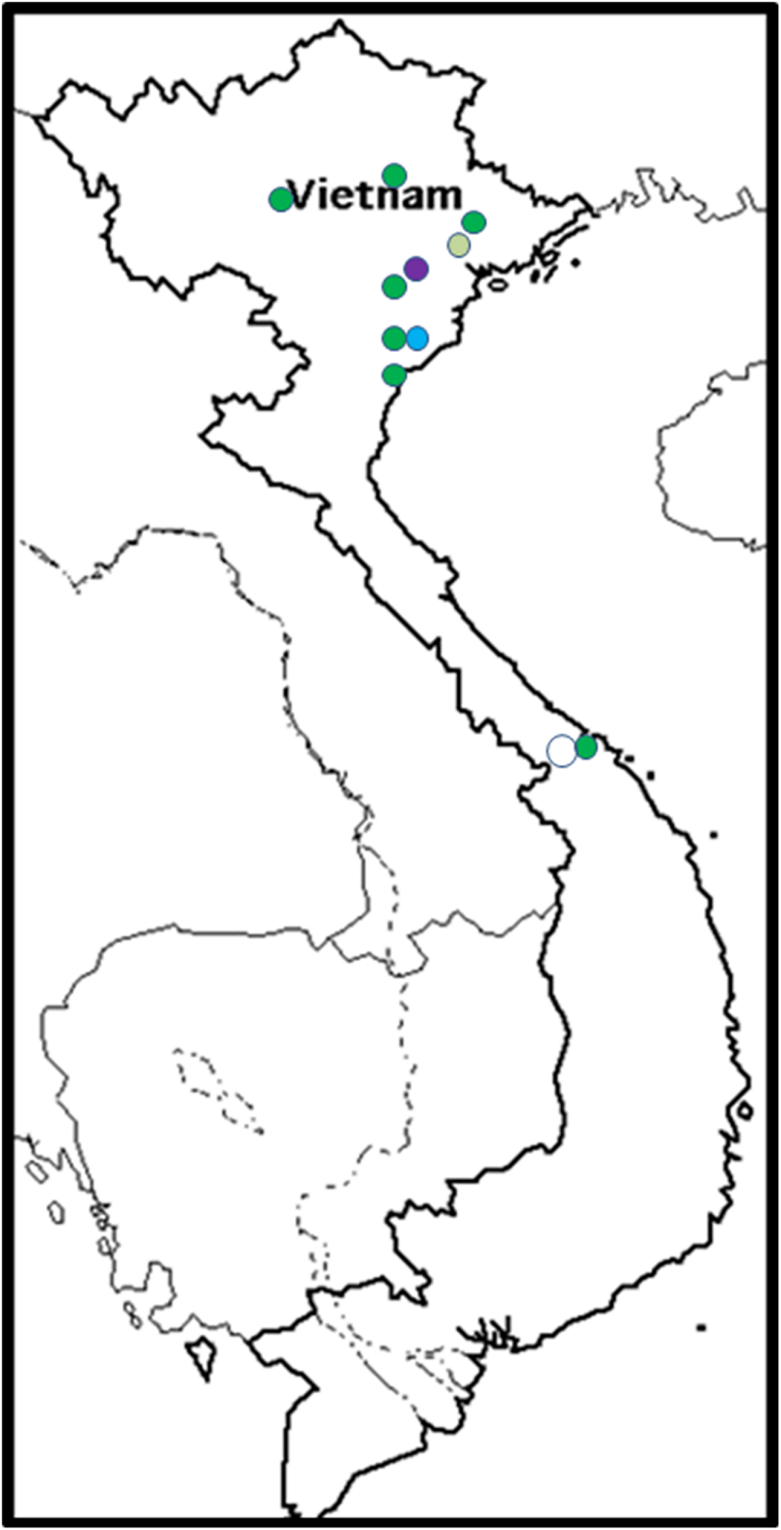
The distribution of *Fusarium oxysporum* f. sp. *cubense* (Foc) vegetative compatibility groups (VCGs) in Vietnam. VCG 0123 is shown in light green, VCG 0124/5 is shown in dark green, VCG 0128 is shown in blue, VCG 01221 is shown in white and VCG 0124/22 is shown in purple.

Five VCGs were found in Vietnam, all members of Clade B, with the exception of VCG 01221. The VCG 0124/5 complex dominated the Vietnamese Foc populations (Fig 1), with isolates collected from all cultivars affected by Fusarium wilt. These included the two Pisang Awak cultivars Chuoi Tay and Chuoi Su (ABB), a Bluggoe cultivar called Chuoi Ngop (ABB) and a Silk cultivar called Chuoi Goong (AAB) (Table 2). VCG 0123 was associated with Chuoi Goong and VCG 0128 with Chuoi Tay. Both were isolated in the north of the country, while VCG 01221 was isolated from Chuoi Su in the central part of the country (Fig 10). Most of the Foc isolates were obtained from Pisang Awak, including VCGs 0124/5, 0128 and 01221 (S1 Table).

### Host association with Foc VCGs

VCG 01213/16 was isolated from *Musa* cultivars of the diploid genotypes AA and the triploid genotypes AAA, AAB and ABB (Table 2). Cultivars within genotype AAA were infected with VCGs 0120/15, 0121, 0122 and 01213/16; all VCGs that belong to Clade A. Exceptions were a single isolate of VCG 0124/5 from Berangan in Malaysia (CAV 2282), one isolate of VCG 0123 from Grande Naine in the Philippines (CAV 1868), several isolates of VCG 0124/5 collected from Gros Michel in Sri-Lanka (S1 Table), and VCGs 0123 and 01221 from Brazilian in China [37]. ABB cultivars were infected by most of the VCGs (VCGs 0120/15, 0121, 0123, 0124/5, 0126, 0128, 01213/16, 01217, 01218, 01219, 01220, 01221 and 0124/22), followed by genotype AAB cultivars (VCGs 0121, 0123, 0124/5, 01213/16, 01217, 01218, 01220 and 0124/22). The diploid genotype AB was associated with the Clade B VCGs 0124/5, 0128, 01217 and 01220. Genotype BB was associated with VCG 0128.

Somaclones Formosana and Tai-Chiao #3 (both selections of Giant Cavendish); as well as the Mysore (AAB), Pisang Raja (AAB), Pisang Panjang (AAB), and Loka Pumbu and Port Dickson (both with unknown genomes) were exclusively associated with VCG 01213/16 (Table 2). The AAB cultivars Mortaman (India), and Bangla Kola (Bangladesh) and the ABB bananas Pisang Awak (Sri Lanka) and Chuoi Ngop (Vietnam) were exclusively associated with VCG 0124/5. The Pisang Awak cultivars Kluai Namwa (ABB) in Cambodia and Guangfen (ABB) in Mainland China were associated with seven known VCGs, respectively (Table 2). Cultivars from which *F*. *oxysporum* isolates were isolated that did not fit into any existing Foc VCGs included Guangfen from China, Plantain and Sabri from Bangladesh, Pisang Kapas, Pisang Awak, Berangan, Mas, Rastali and Plantain in Malaysia, Raja, Manih, Berangan, Cavendish and Kepok in Indonesia [58], Hill Banana, Rastali, Malbhog, Manohar, Ney Poovan and Monthan in India, Red and Silk in Sri Lanka, Namwa in Cambodia, Lakatan and Latundan in the Philippines, Gros Michel, Red and Silk in Sri-Lanka, Namwa and Pei-Chiao in Taiwan and Chuoi Gong and Chuoi Ray in Vietnam (S1 Table).

## Discussion

The banana Fusarium wilt fungus Foc is particularly diverse within the *F*. *oxysporum* species complex when compared to other *formae speciales*. It consists of three races and 24 VCGs, which are substantially more than the number of VCGs found in other important Fusarium wilt fungi such as *F*. *oxysporum* f. sp. *lycopersici*, *F*. *oxysporum* f. sp. *vasinfectu*m, *F*. *oxysporum* f. sp. *melonis* and *F*. *oxysporum* f. sp. *canariensis* [32]. This large diversity could be attributed to the polyphyletic nature of Foc [47], its possible history of sexual reproduction [59], parasexuality [60, 61] and horizontal gene transfer [62]. Vegetative compatibility measures have been successfully employed to determine the origin and global distribution of Foc [28, 63]. The current study, combined with recent publications on Foc diversity in Asia [37, 45, 58], showed the presence of 17 VCGs, thereby confirming the region as the most likely origin of the fungus [11, 29]. VCG diversity in Cambodia, Vietnam and Bangladesh was reported for the first time. The VCGs reported in this study had been identified in Asia before [8, 9, 11, 28, 32, 40, 42, 44, 47, 48, 64], but VCGs 01217, 01221 and 01222 are reported in new countries. VCG 01217 from Bangladesh, Cambodia and Sri Lanka, VCG 01221 from Cambodia and Vietnam and VCG 01222 in India, Bangladesh, Cambodia and Vietnam, were reported from the region for the first time. Sixty-nine *F*. *oxysporum* isolates collected from diseased banana plants also did not fit into any known VCGs. Non-compatible isolates warrant further investigation, as they may represent novel VCGs/populations of this this pathogen.

Two VCG complexes dominate in Asia. The VCG complex 0124/5 was most common in the Indian subcontinent, Vietnam, Thailand and Cambodia, whereas the VCG complex 01213/16 was dominant in the rest of Asia. Their distribution can most likely be attributed to their host range and the movement of planting materials within and between countries. VCG 01213/16, for instance, is well-established in areas where Cavendish bananas are grown in monoculture, such as the Philippines, mainland China and Taiwan. The distribution of this VCG within these countries was mostly associated with the planting of Cavendish bananas. For instance, in the Philippines VCG 01213/16 was found in the Mindanao Island, where 99% of the country’s Cavendish bananas are grown, but not in the Visayas and Luzon where other varieties are grown. Most of the bananas in mainland China and Taiwan are also Cavendish cultivars. In Malaysia and Indonesia, however, VCG 01213/16 is also established on banana varieties other than Cavendish [14, 16]. It is surprising that VCG 01213/16 had not spread from Malaysia and China into neighbouring Thailand, Cambodia and Vietnam, as many cultivars grown in the latter countries are also susceptible to this strain [9, 21]. Perhaps the mixed crops and banana cultivars that are used in these countries do not favour its establishment. The recent expansion of the Cavendish banana industry in Vietnam and Thailand [5] may increase the risk of an outbreak of Fusarium wilt caused by VCG 01213/16 in this area.

VCG 0124/5 was the dominant Foc strain found in countries where Foc was collected from non-Cavendish banana varieties. This strain was present in all Asian countries, apart from the Philippines. This could be due to a sampling bias, as most of the samples collected in the Philippines were from Cavendish bananas. VCGs within Foc Lineage VI, which include VCG 0124/5/8/12/20/22, are some of the most widely distributed in the world and occur in all regions where banana are cultivated [9, 14, 24, 25, 28, 29, 30, 46, 63]. The widespread occurrence could be due to human dispersal of planting material. VCG 01220, for example, which is commonly found throughout the Indian subcontinent, is also found in East and Central Africa (ECA) [65], a region with a well-established relationship with India. Fusarium wilt was discovered in ECA when a large community of Indian emigrants settled in the area soon after World War II ended [66]. It is possible that VCG 01220 and other Lineage VI VCGs could have been introduced into the region by Indian settlers.

India is the largest producer of bananas in the world. More than 20 banana varieties are grown commercially in the country, and the fruit harvested is mostly intended for the domestic market. More than half of India’s bananas are Cavendish types. Other popular varieties include Ney Poovan, Rasthali, Nendran, Bluggoe and Pome. Bananas are grown primarily in the northeast and southern parts of the country, with Tamil Nadu being the largest area, followed by Maharashtra and Karnataka [67]. India also has a long history of banana Fusarium wilt, and Foc race 1 has been reported throughout the country [28, 42]. In the current investigation, three VCGs (VCGs 0124/5, 0128 and 01220) were found on Ney Poovan, Silk, Monthan and Pisang Awak bananas in southern India. Despite the significant Cavendish industry, no Foc race 4 VCGs, such as VCG 0120/15 and VCG 01213/16, were detected. An unusual association between VCG 0124 and Cavendish bananas, however, was reported by Thangavelu *et al*. (2012) [68] following a survey conducted in the Tamil Nadu region. Cavendish bananas are considered immune to Foc race 1 and was employed to control the Fusarium wilt epidemic on Gros Michel bananas in Central America during the 1960s. With the exception of that caused by VCG 01213/16, damage to the Cavendish bananas requires predisposing factors; in the latter cases, there is significant genotype x environment (GxE) interactions [51].

GxE interactions may be responsible for some unexpected results in the current investigation. A single association occurred between Brazilian cultivar (AAA Cavendish) and VCG 0123 (regarded as Foc race 1) in China. VCG 0123 has been associated with Grande Naine before [69], but as a rule are usually associated with cultivars susceptible to Foc races 1 and 2. In Indonesia an isolate of VCG 01213/16 was isolated from the AA cultivar Rose (synonym Rejang) [58], which were previously reported to be resistant [70]. The most unusual association between VCG and banana variety was the single isolate from VCG complex 0124/5 on Berangan (AAA) in Malaysia. These associations may be indicators of the environmental contribution to the outcome of these interactions.

Several Foc VCGs were not found in Asia. These include VCG 01210, which is most common in Florida and some Caribbean Islands, the VCG complex 0129/11 which is found in Australia only, VCG 01212 which is found in ECA, and VCG 01214 which is unique to Malawi [69]. This study reports VCGs in new countries for the first time. VCG 01221 was first reported from Thailand [46], and was now also found in Cambodia, Vietnam and mainland China. VCG 01222 was first reported from Malaysia, and thereafter from Uganda, where it frequently formed a complex with VCG 0124 [71, 72]. In the current study, VCG 01222 were also found in India, Bangladesh, Cambodia and Vietnam. VCG 01223 and 01224 were not isolated from banana samples collected in this study. It was originally isolated from Pisang Keling and Pisang Ambon in Malaysia, respectively [33]. Since isolates recovered from these cultivars in Malaysia were limited, future surveys could reveal a more widespread occurrence.

A number of *F*. *oxysporum* isolates associated with banana did not fit into any known VCG, despite being self-compatible. These isolates might either be non-pathogenic endophytes, or putative novel Foc VCGs not previously detected. Another seven isolates described by Riska and Hermanto (2012) [58] were also not compatible to known VCGs. Bentley *et al*. (1998) [28] found 13 putative VCGs or genotypes when investigating a worldwide population of Foc. These incompatible isolates should be tested for pathogenicity on banana, vegetative compatibility, and relatedness to previously described genotypes of the pathogen.

VCG complexes were frequently observed in the current study, including cross compatibility between VCGs 01213 and 01216; 0124 and 0125; 0124 and 01222 respectively. Complexes in Foc VCGs have been reported before, and often involve two or more VCGs [7, 24, 28, 25, 29, 46, 60, 62]. VCGs are usually identified by heterokaryons that form between individuals with common alleles at their *vic* loci [57]. A mutation at a single *vic* locus, thus, could place closely related individuals in different VCGs [28, 31]. When unknown strains are characterized, they could then pair with either or both VCGs, resulting in cross compatibility between different VCGs. The VCGs within complexes, however, are all closely related when genetic distances are measured [29]. It might be more prudent to regard these VCG complexes as single taxonomic units.

An understanding of the diversity of Foc in banana countries and regions can have important management implications. It could guide quarantine authorities on the movement of planting materials, and allow growers in the deployment of resistant varieties. However, the success of planting resistant varieties against Foc VCGs present in Asia should not be based solely on the findings of the current study. The collections made in this investigation in some instances were geographically localised and with sampling bias, and thus only provided a representation of Foc diversity in the region. For instance, in Vietnam there were no collections made in the southern region. Future studies will need to be more representative of regions and cultivars. Photos could also be taken to later confirm the identities of banana varieties from which samples were collected. Varietal reactions will also have to be field tested against all races and VCGs of Foc present in the area. This involves the inclusion of control varieties and a proper statistical layout to ensure uniform and sufficient disease development.

## Supporting information

S1 Table Morphological identity, PCR-RFLP clade and lineage identity, vegetative compatibility group, host and host subgroup, location and origin of *Fusarium* isolates collected in Asia. (XLSX)

S2 Table Vegetative compatibility tester strains used to characterize compatibility to Asian *Fusarium oxysporum* f. sp. *cubense* isolates. (XLSX)
